# BHV-1 induced oxidative stress contributes to mitochondrial dysfunction in MDBK cells

**DOI:** 10.1186/s13567-016-0332-2

**Published:** 2016-03-22

**Authors:** Liqian Zhu, Chen Yuan, Dong Zhang, Yan Ma, Xiuyan Ding, Guoqiang Zhu

**Affiliations:** College of Veterinary Medicine and Jiangsu Co-innovation Center for Prevention and Control of Important Animal Infectious Diseases and Zoonoses, Yangzhou University, 48 Wenhui East Road, Yangzhou, 225009 China; The Test Center of Yangzhou University, 48 Wenhui East Road, Yangzhou, 225009 China

## Abstract

The levels of cellular reactive oxygen species (ROS) and ATP as well as the mitochondrial membrane potential (MMP) in response to bovine herpesvirus 1 (BHV-1) infection of MDBK cells were measured, respectively. BHV-1 infection increased ROS production which depended on viral entry, and de novo protein expression and/or DNA replication. Vice versa, excessive ROS was required for efficient viral replication. Levels of both ATP and MMP were significantly decreased after BHV-1 infection. Interestingly, the loss of MMP was ameliorated by ROS depression. Collectively, ROS dependent mitochondrial damage and ultimately disruption of energy metabolism (ATP depletion) are a potential pathogenic mechanism for BHV-1 infection.

## Introduction

Bovine herpesvirus 1 (BHV-1), an enveloped virus belonging to the *alphaherpesvirus* subfamily, infects cattle of all ages and breeds worldwide. Acute infection of BHV-1 results in clinical diseases in the upper respiratory tract, nasal cavity, or ocular cavity [1, 2]. Generally, BHV-1-induced immune suppression initiates a polymicrobial respiratory tract disease, commonly referred to bovine respiratory disease complex (BRDC), which costs the US cattle industry approximately three billion dollars, annually [2].

Reactive oxidative species (ROS) such as superoxide, hydrogen peroxide (H_2_O_2_), peroxynitrite (OONO^−^) and hydroxyl radicals (OH^.^) have been shown to play important roles in both physiological and pathophysiological processes. Under physiological conditions, the production and release of ROS is tightly controlled at specific time and space, which broadly influences cellular processes such as gene expression, cells proliferation, migration and differentiation [3]. Oxidative stress results from the imbalance between production of ROS and the protective effect of the antioxidant system responsible for their neutralization and removal [4]. And excessive production of ROS generally inflicts cell damage through oxidation of macromolecules, such as protein and lipids [5]. Increasing studies suggested that host ROS contributed greatly to the replication of numerous viruses, as well as inflammation-associated tissues damage, such as in the inflammatory diseases caused by influenza virus, Hepatitis C virus and Dengue virus [6–10]. Thus, suppression of ROS appears to be a potential therapeutic approach primarily through reduction of both viral burden and inflammatory response.

It has been demonstrated that BHV-1 infection of MDBK cells induced apoptotic responses through diverse mechanisms including the mitochondria-mediate pathway [11, 12]. And mitochondrial damage is a potential indicator of cell apoptotic response. To our knowledge what happed to the mitochondria in BHV-1 infected MDBK cells is unknown.

In this study, we demonstrated that high level of cellular ROS was dramatically induced due to BHV-1 virus entry, de novo protein expression and/or DNA replication. The increased ROS was a crucial cellular signaling contributed to viral replication and mitochondrial damage, which consequently interfered with cellular ATP production. Our results suggested that the enhanced ROS production by BHV-1 infection is a double-edged sword, which not only promoted the viral infection, but also brought damage to the cells due to the injury to mitochondrial as well as ATP depletion. This could be a potential mechanism for the pathogenicity of BHV-1 infection induced tissue damage.

## Materials, methods and results

**Figure 1:**
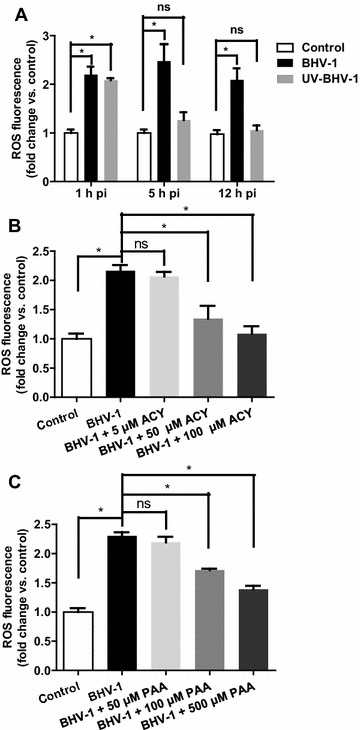
**Intracellular ROS was increased following BHV-1 infection.** **A** MDBK cells were infected with UV-inactivated or untreated BHV-1 at MOI of 10. At indicated time point, H2DCFDA (5 μM, 30 min) was loaded for visualization of ROS with fluorescence microscopy. **B** and **C** MDBK cells pretreated with inhibitor ACY (**B**) or PAA (**C**) at indicated concentrations were infected with BHV-1 (MOI = 10) in the presence of corresponding inhibitor. At 4 h pi intracellular ROS were detected with H2DCFDA. Quantification of fluorescence intensity was performed using Image-pro Plus 6. The measured fluorescence intensity were expressed as a relative fold change vs. that in the control cells. Data represent three independent experiments with triplicates performed at each time. Statistical analyses were performed using Student’s *t* test (Asterisk indicates *P* < 0.05 vs. control).

To evaluate whether BHV-1 infection triggered intracellular ROS production, MDBK cells (kindly provided by Dr Leonard J. Bello, University of Pennsylvania) were infected with BHV-1(Colorado 1 strain, kindly provided by Dr Leonard J. Bello) (MOI = 1) at 37 °C for 1, 5 and 12 h. We selected these time points because BHV-1 replication starts within 2 h of infection in cattle, with cell surface antigen expression within 3–4 h after infection and viral release and spread starting 8 h after infection [13]. For the control, the cells were mock infected with supernatant of cell cultures that were made by frozen-thawing and subsequent centrifugation using a procedure similar to the generation of virus stocks. The level of intracellular ROS was determined using ROS fluorescence indicator 2′,7′–dichlorodihydrofluorescein diacetate (H2DCFDA) (Sigma-Aldrich, St. Louis, MO, USA) which can be converted to fluorescent compound dichlorofluorescein (DCF). The images were acquired under a fluorescence microscope (Olympus BX-51, Olympus, Tokyo, Japan), and fluorescence intensity was quantified using Image-Pro Plus 6 software. Compared to the control, BHV-1 infection of MDBK cells indeed significantly increased the production of intracellular ROS at all the detected time points (Figure 1A). Since cellular ROS was significantly increased by the viral infection at 1 h post-infection (pi), ultraviolet (UV)-inactivated viral particles, a replication deficient virus that could enter cells but could not finish subsequent replication steps, was employed to elucidate whether viral entry process increase cellular ROS. The (UV)-inactivated viral particles showed similar capacity to that of live virus to induce ROS at 1 h pi, but it declined to a level near the mock infected control at 5 and 12 h pi (Figure 1A).

Acyclovir (ACY) is a known inhibitor to suppress herpesviruses DNA synthesis [14]. The relation between BHV-1-induced ROS production and viral DNA synthesis was investigated through treatment of MDBK cells with ACY. MDBK cells were pretreated with ACY (Sigma-Aldrich, St. Louis, MO, USA) at concentrations ranged from 100 to 5 mM for 1 h, and then infected with BHV-1 at MOI of 10 along with the inhibitor. At 4 h pi the cellular ROS was detected with H2DCFDA. As a result, cellular ROS induced by viral infection was significantly depleted by ACY with a dose-dependent manner (Figure 1B). Phosphonoacetic acid (PAA), is a more specific inhibitor targeting the viral DNA polymerase [15]. Treatment of MDBK cells with chemical PAA (Sigma-Aldrich) at concentrations ranged from 500 to 50 μM with the same manner as that with ACY led to a reduction of viral induced ROS with a dose-dependent manner (Figure 1C). It is correlated with the result of MDBK cells treated with ACY. Thus, de novo viral protein production and/or DNA replication seems to be correlated to the elevated ROS levels, which was further confirmed by the analysis with UV-inactivated viral particles.

**Figure 2:**
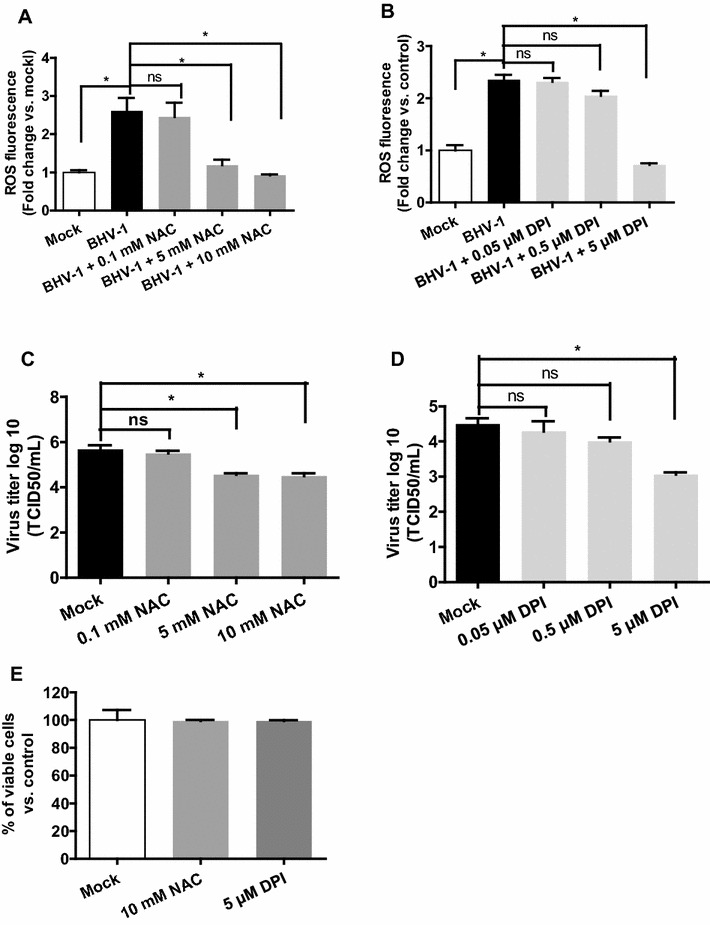
**The reduction of ROS levels by both ROS scavenger NAC and NOXs inhibitor DPI inhibited BHV-1 replication in MDBK cells.** (**A** and **B**) MDBK cells subjected to a pretreatment with DMSO control or chemicals NAC (**A**) or DPI (**B**) for 1 h, were mock infected with supernatant of cell culture or infected with BHV-1 along with chemical treatment. At 4 h pi cellular ROS were detected using H2DCFDA (5 μM, 30 min), and the quantification of fluorescence intensity was performed using Image-pro Plus 6. (**C** and **D**) MDBK cells infected with BHV-1 (MOI = 1) were treated with chemicals including NAC (**C**) or DPI (**D**) during viral infection plus a pretreatment for 1 h. At 24 h pi the virus yields were determined with TCID_50_ method. (**E**) Cell viability of MDBK cells exposed to each inhibitor as designated concentrations was detected with MTT assay. Values represent three independent experiments. Statistical analyses of significant difference vs. mock were performed using Student’s *t* test (Asterisk indicates *P* < 0.05 vs. mock).

NADPH oxidases (NOXs) are major sources of ROS production, which are implicated in multiple viral infections, such as in influenza virus infection [8, 16]. Here, the widely used NOX inhibitor diphenylene iodonium (DPI) and ROS scavenger N-Acetyl-l-cysteine (NAC) were employed to assess the effect of cellular ROS on BHV-1 replication in MDBK cells. These cells were pretreated with DPI (Sigma-Aldrich) or NAC (Sigma-Aldrich) at indicated concentrations for 1 h, and then infected with BHV-1. At 4 h pi and 24 h pi cellular ROS and viral titer were detected, respectively. The treatment of MDBK cells with both DPI and NAC at indicated concentrations resulted in significant reduction of BHV-1-induced cellular ROS as well as the progeny virus yield with a dose-dependent manner, respectively (Figures 2A–D). The compound at the tested concentrations didn’t show apparent cytotoxicity to MDBK cells as determined by MTT (3-[4,5-dimethylthiazol-2-yl]-2,5 diphenyl tetrazolium bromide, provided by Sigma-Aldrich) assay performed as we previously described [17] (Figure 2E). It ruled out the possibility that reduced virus titer was caused by strong cellular toxicity of chemicals. Obviously, the increased cellular ROS was required for BHV-1 replication.

**Figure 3:**
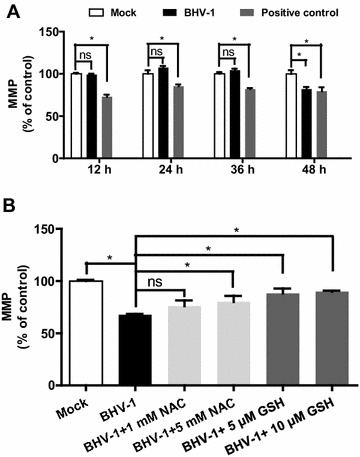
**Effect of BHV-1 on mitochondrial membrane potential in MDBK cells.** (**A**) MDBK cells of confluent in 96-well plates were infected with BHV-1 at MOI of 0.01 for indicated time length, and then MMP was detected with JC-1 dye. The treatment of cells with CCCP for 20 min was used as a positive control. (**B**) MDBK cells of confluent in 96-well plates were treated with NAC and GSH at indicated concentrations, before and during virus infection with BHV-1 (MOI = 0.01) for 48 h. The MMP was detected with JC-1 dye. Data represent three independent experiments. Statistical analyses were performed using Student’s *t* test (Asterisk indicates *P* < 0.05 vs. mock).

To assess if BHV-1 infection caused mitochondrial dysfunction, MDBK cells of confluent in 96-well plates were mock infected with DMEM medium containing cell lysis or infected with BHV-1 (MOI = 0.01). Carbonyl cyanide-m-chlorophenyl hydrazone (CCCP) is a known chemical causing rapid mitochondrial depolarization  [18]. Here, CCCP (Beyotime Biotechnology, Jiangsu, China) was introduced to treat the cells as a positive control. At 12, 24, 36 and 48 h pi, the cells were washed twice with PBS, incubated with 3 μg/mL of 5,5′,6,6′-Tetrachloro-1,1′,3,3′-tetraethylbenzimidazolyl-carbocyanine iodide (JC-1) (Beyotime Biotechnology, Jiangsu, China) for 20 min at 37 °C. JC-1 dye is a widely used indicator of mitochondrial polarization [19, 20]. In healthy cells with higher mitochondrial potential, JC-1 forms a red-fluorescent aggregate (dimer). In unhealthy cells with lower mitochondrial membrane potential, JC-1 remains in the cytoplasm as green—fluorescent monomers resulting in a decrease in the red/green ratio. The decrease in the ratio of red/green fluorescence at 48 h pi following BHV-1 infection indicated the loss of mitochondrial membrane potential (MMP), which showed a level similar to the positive control treated with 1 μM of CCCP (Figure 3A).

In view of that overproduction of cellular ROS may exhibit toxic effect on the mitochondria, MDBK cells were treated with both NAC and Glutathione (GSH, provided by Beyotime Biotechnology, Jiangsu, China), a main intracellular non-enzymatic antioxidant exerting an efficient buffering role against ROS during the virus infection, to characterize the relation between ROS and the loss of MMP. As a result, both NAC (at a concentration of 5 mM) and GSH (at concentrations of 5 μM and 10 μM) significantly ameliorated the drop of MMP induced by BHV-1 infection at 48 h pi (Figure 3B). It implied that the virus triggered overproduction of ROS was partially associated with the loss of MMP.

**Figure 4:**
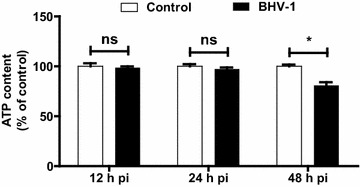
**Effect of BHV-1 on ATP content during the infection of MDBK cells.** MDBK cells of confluent in 6-well plates were infected with BHV-1 at MOI of 0.01 for indicated time length. The content of ATP and protein concentration was determined with ATP Assay Kit and Bradford Protein Assay Kit, respectively. The ATP content was normalized to the protein concentrations and expressed as a % of mock treated control cells. Data represent three independent experiments. Statistical analyses were performed using Student’s *t* test (Asterisk indicates *P* < 0.05 vs. control).

Because ATP is mainly produced in the mitochondria which is associated with energy metabolism, the loss of ATP content is considered to be a powerful indicator of mitochondrial dysfunction. Confluent MDBK cells in 6-well plates were mock infected with DMEM containing supernatant of cell culture or infected with BHV-1 (MOI = 0.01). At 12, 24 and 48 h pi, ATP content was determined with a commercial luminescence kit (Beyotime Biotechnology, Jiangsu, China), the intensity was read with multifunctional Microplate Reader (SpectraMax M2, MDC) and normalized to the respective protein concentrations in the cell lysis detected with Bradford Protein Assay Kit (Beyotime Biotechnology, Jiangsu, China). Interestingly, comparing to the control, ATP content was significantly decreased at 48 h pi in MDBK cells (Figure 4), which correlated well with the reduction of MMP by BHV-1 infection (Figure 3A). It further confirmed that the virus infection led to mitochondrial dysfunction.

## Discussion

Overproduced cellular ROS or oxidative stress is a critical factor that influences disease outcome, in the case of multiple viral infection such as by infection of influenza virus and Hepatitis C virus [6, 8, 21]. In this study, we found that BHV-1 infection of MDBK cells dramatically increased the production of cellular ROS, which is essential for virus replication (Figures 1 and 2).

BHV-1 together with Herpes simplex virus (HSV) and HSV-2 all belongs to the *Alphaherpesvirinae* subfamily, and shares a number of biological properties. The interplays between ROS and HSV-1 or HSV-2 in various cell cultures have been extensively studied [22–24]. Based on both our studies and studies from others [23–25], we inferred that cellular ROS is broadly regulated by alpha herpesviruses, but diverse manners were presented, which are cell type and virus specific, e.g., HSV-2 induced ROS production in RAW246.7 cells could be uniquely detected at 1 h pi [25], HSV-1 infection of murine microglial cells and neural cells increased ROS levels at time durations of 24–72 and 1–24 h pi, respectively [23, 24]. Increased ROS levels in MDBK cells was detected at 1–12 h pi during BHV-1 infection (Figure 1), and also it appeared at 24 h pi (data not shown). It is highly possible that ROS have diverse effect on viral replication or even on viral pathogenesis.

ROS mainly originated from Nox family of NADPH oxidases and mitochondria. Mitochondria are also susceptible to insult from ROS, since both mitochondrial proteins and mitochondrial DNA are vulnerable to ROS [26]. It is known that mitochondria is a potential target of oxidative stress for environmental toxicants stimulus like the fine particulate matter (PM2.5) [27]. It is reasonable that mitochondria is a potential target by some viruses that are able to induce excessive ROS. It has been documented that HSV infection results in the loss of MMP and decreases the levels of cellular ATP at the late stage of infection [28], whereas the role of ROS in this adverse effect is not yet elucidated. In this study, we found that BHV-1 infection of MDBK cells rendered mitochondria dysfunction as demonstrated by the mitochondrial depolarization and reduced ATP levels at late stage of infection. The loss of MMP was partially reversed by antioxidants of GSH and ROS scavenger NAC, which indicated that the overexpressed ROS partially accounts for the mitochondrial dysfunction.

Currently, the pathogenic mechanism of ROS in viral disease mainly reside in the ROS—mediated promotion of viral replication and inflammatory response. We suggested that BHV-1 induced ROS/mitochondrial damage and ultimately interfered with energy metabolism (as demonstrated by ATP depletion) is a novel mechanism by which BHV-1 induced cell injury. In addition to ROS mediated inflammatory response, this is also a potential pathogenic mechanism employed by alpha herpesvirus infection, such as in BHV-1 infection.
